# Longitudinal comparison of bacterial pathogen seropositivity among wet market vendors in the Lao People’s Democratic Republic

**DOI:** 10.1016/j.onehlt.2023.100618

**Published:** 2023-08-25

**Authors:** Nilandone Senvanpan, Vilayouth Phimolsarnnousith, Sayaphet Rattanavong, Mayfong Mayxay, Daniel Reinharz, Amanda E. Fine, Paul F. Horwood, Philippe Dussart, Stuart D. Blacksell, Mathieu Pruvot, Paul N. Newton, Matthew T. Robinson

**Affiliations:** a Institute de la Francophonie pour la Médecine Tropicale (IFMT)/Lao Tropical & Public Health Institute, Vientiane, Lao PDR; b Lao-Oxford-Mahosot Hospital-Wellcome Trust Research Unit (LOMWRU), Microbiology Laboratory, Mahosot Hospital, Vientiane, Lao PDR; c Centre for Tropical Medicine and Global Health, Nuffield Department of Medicine, University of Oxford, Oxford, UK; d Institute of Research and Education Development (IRED), University of Health Sciences, Ministry of Health, Vientiane, Lao PDR; e Département de Médecine sociale et préventive, Université Laval, Québec, Canada; f Wildlife Conservation Society, Health Program, 2300 Southern Blvd, Bronx, NY, 10460, USA; g Virology Unit, Institut Pasteur du Cambodge, Pasteur Network, Phnom Penh, Cambodia; h Mahidol-Oxford Tropical Medicine Research Unit (MORU), Faculty of Tropical Medicine, Mahidol University, Bangkok, Thailand; i University of Calgary, Faculty of Veterinary Medicine, Calgary, AB, Canada

**Keywords:** Wildlife meat, markets, Laos, zoonoses, rickettsia, scrub typhus, leptospira, murine typhus

## Abstract

Wild animal trade for human consumption is a global issue, involving complex interactions between economics, culture, food security and conservation. Whilst being a biodiversity issue, it is also a major public health concern, with recent epidemics and pandemics of zoonotic pathogens linked to interactions with wildlife. At three time points, between March 2017 and June 2018, a longitudinal sero-survey of 150 market vendors from three wet markets in Laos (selling vegetables, domestic animal meat and/or wildlife meat) was conducted to determine if vendors had been differentially exposed to three endemic bacterial pathogens – *Orientia tsutsugamushi, Rickettsia typhi*, and *Leptospira* spp. A total of 367 serum samples were tested by IgG enzyme-linked immunosorbent assay (ELISA) and immunofluorescence assay (IFA, for scrub typhus group (STG) and typhus group (TG) only). Among vendors, 32.7% were IgG-positive for at least one pathogen, 13.3% sero-converted during the study. Multi-season occupancy modelling for STG indicated a significantly higher prevalence of STG IgG in vegetable vendors (27.3%) and wildlife vendors (28.4%) than in domestic animal meat vendors (6.9 %, p=0.05), and higher in Phonsavanh market (OR=9.6, p=0.03) compared to Lak Sao and Salavan markets. Estimated mean incidence was 57 cases per 10,000 per 7.5-month period. For TG, vendor age had a significant effect on prevalence (OR=1.04, p=0.006), estimated mean incidence was 64 cases per 10,000 per season (7.5-month period). Despite individuals selling domestic meat having a higher prevalence of *Leptospira* infections than those that did not (11.6% versus 4.5%), the difference was not significant. Whilst this study has a number of limitations, including vendors changing what food types they sold and no investigation of exposure outside of markets, the finding that the risk of exposure of vendors to zoonotic pathogens may be associated with types of food sold for human consumption warrants further investigation.

## Introduction

The hunting, trading and consumption of wild animals and wild meat is a global concern, involving complex interactions between economics, cultural traditions, food security and conservation [1]. Whilst Southeast Asia is recognised as a hotspot for illegal wildlife trade [2, 3], data on the consumption of wildlife meat and its public health impacts are limited. Studies have suggested that illegal wildlife trade in Vietnam, including wildlife meat consumption, generates an annual revenue of $67 million [4], whilst in Sarawak, Malaysia, approximately 23,500 tonnes of wildlife meat is eaten annually [5]. Estimates in the Lao People’s Democratic Republic (Laos) suggests average market trades over 71 tons of wildlife meat annually, generating nearly US$500,000 for the local economy [1]. The types of wild animals sold in wet markets in Laos are variable, with squirrels being the most frequently reported purchased animal type (59%)[1, 6]. In Laos, consumers came from diverse socioeconomic backgrounds, and mainly described their reasons for purchasing wildlife meat as linked to the perception that wildlife meat was healthier, and tasting better than domestic animal meat [1].

The interactions between humans and animals in the process of hunting, selling and consuming wildlife meat raises public health concerns. Zoonotic pathogens account for 61% of pathogenic organisms that may infect humans [7] and are responsible for the majority of emerging infectious disease events, which are significantly increasing in occurrence [8, 9]. It has been estimated that endemic and emerging zoonotic diseases account for 26% of the disability-adjusted life years (DALYs) lost to infectious disease and 10 % of the total DALYs lost in Low- and top-Income Countries (LMICs)[10]. Although zoonotic viruses pose a great risk, as seen with Ebola, SARS-CoV and SARS-CoV-2 [11–13], over half of the pathogens involved in emerging infectious disease events are bacterial [8], with a key group being the Rickettsiales. Rickettsial organisms are endemic in Laos, with reports identifying 27% of patients with non-malarial febrile illness having a rickettsial infection [14]. There is little information on risks of zoonotic pathogen transmission in markets, especially for bacterial pathogens. In Laos, 50.6% of consumers perceived some risk from consuming or handling wildlife meat, with 35.2% agreeing that animals could transmit disease [1]. In contrast, only 23% of wildlife meat vendors said their produce could transmit disease, and 86% did not consider their health at risk from their occupation [15]. A PCR survey of 324 wild animals found at wet markets identified 21.3% positive for at least one bacterial pathogen, including *Leptospira* spp. (the most frequent pathogen, in 20.1% of animals), *Rickettsia* spp. (including *R. typhi, R. felis*, and *R. conorii)*, Anaplasmatace, *Anaplasma phagocytophilum* and *Ehrlichia chaffeensis* [6].

To understand the risks associated with exposure and handling of wildlife meat by vendors, we carried out a one-year longitudinal serosurvey of market vendors to characterise the frequency of seropositivity and seroconversion to three common endemic zoonotic bacterial pathogens in Laos: *Rickettsia typhi*, the agent of murine typhus, *Orientia tsutsugamushi*, the agent of scrub typhus, and *Leptospira* spp. We hypothesized that wildlife vendors would have increased exposure to these pathogens, as measured by an increase in rates of sero-conversion compared to other vendor types.

## Methodology

Wet markets in the provincial towns of Phonsavanh (Xieng Khouang Province), Lak Sao (Bolikhamxay Province) and Salavan (Salavan Province) were selected as these were known for high wildlife meat availability [1, 15, 16] . All vendors selling either vegetables, domestic animal meat (i.e.: pig, chicken, beef or buffalo), or wildlife meat (and combinations thereof) were invited to participate in Survey-1 in March/April 2017 (dry season). After the study was explained in Lao language, informed written consent was obtained and the questionnaire was administered in Lao language (Supplementary material). When necessary, a translator was used to discuss the consent and questionnaire in other local languages. Venous blood (5ml) was collected and allowed to clot to obtain serum. Participants were offered free blood pressure and glucose checks. Questionnaire and blood sampling were repeated twice over a period of one year on the same vendors, with Survey-2 in September/October 2017 (end of monsoon), and Survey-3 in May/June 2018 (start of monsoon).

Survey-1 samples were tested for the presence of IgG and IgM against typhus group (TG, *R. typhi)*, scrub typhus group (STG, *O. tsutsugamushi)* and *Leptospira* spp. using previously described ELISAs [17–20] and a commercial anti-leptospirosis IgG/IgM ELISA kit following manufacturer recommendations (SERION ELISA classic, Germany). Sera from all three surveys were tested for the presence of IgG against TG, STG and *Leptospira* spp., as above. For TG and STG ELISAs, samples with a net OD ≥0.5 had IgG IFAs conducted to confirm positivity [17–20].

Statistical analyses were carried out using Stata 14 (Statacorp, USA) and R (v4.0.3, R Core Team, Vienna, Austria). Median values and interquartile ranges were calculated where appropriate. Prevalence and binomial confidence intervals were estimated for each pathogen. Effect of vendor type was assessed using Chi-squared (or Fisher Exact) statistics for binary outcomes (e.g. positive/negative) or with ANOVA for continuous variables (e.g. age).

Since ELISA testing was duplicated for the first survey, all results were analysed using an occupancy modelling framework, allowing separate prevalence and detection probability estimates (<100% due to imperfect sensitivity), thereby providing true prevalence estimates [21, 22]. Specifically, a multi-season occupancy model was used, which models the occurrence data as a probability of occupancy (i.e. prevalence) at time t and subsequent time steps (t+1 and t+2) as the probability of positive seroconversion between t and t+1 (or between t+1 and t+2) (parameter gamma, practically a cumulative incidence rate) and negative seroconversion between t and t+1 (parameter epsilon). Candidate explanatory variables included: market, and vendor age, and exposure to different products. Exposure was allowed to be entered into models in two different ways, as a vendor type (consistent with previous definitions), or as separate binary exposure to vegetable, wild meat or domestic animal meat at any time through the study. Because of the significance of squirrels as carrier of the target pathogens [6], we also tested the effect of a squirrel exposure binary variable. Models were fitted using the package RPresence [23] and compared using the Akaike Information Criterion (AIC). *P*-values ≤0.05 were considered significant. This study was approved by the Lao National Ethics Committee of the Ministry of Health, No. 36/NECHR (Submission ID 2017.26. NW).

## Results

For Survey-1, 170 market vendors were approached. Twenty vendors declined to participate - more than half of those were wildlife vendors and were worried about speaking to government representatives likely due to concerns around the legality of trading wildlife. Others cited a dislike to needles or did not want a blood check done. In total, 150 vendors from Xieng Khouang (n=58), Bolikhamxay (n=31) and Salavan (n=61) markets consented to participate, completing the questionnaire and providing a blood sample. For Survey-2, 117 (78%) were successfully followed-up, and 100 (67%) in Survey-3. Reasons for the loss to follow-up included being uncontactable by telephone, participants declining to provide information and blood for the second or third time, or being busy with agricultural harvest. Loss to follow-up was comparable in vegetable vendors (32% at Survey-2 and 54% at Survey-3) and wild meat vendors (23% at Survey-2 and 33% at Survey-3) but greater than domestic meat vendors (11% at Survey-2 and 19% at Survey-3). All vendors were female (100%) with a median age of 38 years (IQR:30-46; n=150); no significant differences in age were seen between markets (F-statistic=0.98, p=0.38) or vendor types (F=0.20, p=0.65). The majority were educated to secondary level (45.3%), whilst 34% were educated to primary level, and 18% self-identified as illiterate; 2.7% attended a college or university. Differences in education level were significant between locations, with Xieng Khouang having a higher proportion of vendors educated to secondary level than Bolikhamxay or Salavan (Chi-squared test p<0.001). No significant differences in education level was seen between vendors types (Chi-squared test p=0.361). There were significant differences between ethnicities of vendors between markets (Fisher Exact test, p<0.001). Whilst the majority (65.3%) identified as Lao Loum ethnicity, proportions of other ethnic groups varied by location. Non-Lao Loum individuals were primarily Hmong in Xieng Khouang and Bolikhamxay, and Ngae in Salavan (Table 1). There were significant differences in ethnicity between vendor types (Fisher Exact test, p<0.001), with differences mainly resulting from a greater representation of Ngae ethnic group as wildlife vendors. The median years spent as a market vendor was 7 (IQR:4-10). When asked in Survey-1, 64.0% of vendors sold vegetables, 36.0% sold domestic animal meat and 36.0% sold wildlife meat. The majority of vendors (64%) sold only one type, whilst the remaining vendors sold combinations of those three; no vendor sold all three types of items. Of those vendors available for repeated surveys (n=119), 50.4% changed what they sold during the study. In total, 70.0% of all vendors sold vegetables, 54.7% sold domestic animal meat and 44.0% sold wildlife meat at some point during the study. Few vendors participating in all three surveys sold only one type of produce, with 16 selling only vegetables and 31 selling only domestic animal meat; none sold only wildlife meat. Therefore, in the rest of the analysis, all vendors were regrouped into the following: vendors only selling vegetables at all points during the study (24.7%), vendors selling domestic animal meat with or without vegetables but no wildlife meat (31.3%), and vendors selling wildlife meat at any point of the study, with or without vegetables and/or domestic animal meat (44.0%).

Of those who responded, 41.4% of vendors who sold domestic or wildlife meat at some point (n=128) said they have been bitten/scratched whilst handling animals. This increased to 61.8% for those currently selling wildlife meat. Of 61 vendors selling wildlife meat, 85.2% kept wild animals at home – particularly when animals were not immediately sold at market (Figure 1). These were primarily squirrels (78.6% of vendors who had animals), wild birds (51.8%), deer (28.6%), civets (26.8%), wild pigs/boar (23.2%), and monitor lizards (23.2%). Only 6% of wildlife vendors hunted, but most butchered the animals themselves (96.9%). Of those who sold domestic animal meat, 80.2% kept domesticated animals at home, including chickens (81.5%), ducks (47.7%) and pigs (40.0%). Of 100 vendors, 72.0% butchered domestic animals at home.

Initial analysis of Survey-1 samples identified detectable IgG and IgM against at least one pathogen in 42.7% (64/150, 95% CI:34.7-51.0) and 18.7% (28/150, 95% CI:13.0-26.0) of vendors, respectively. The proportion of wild meat vendors who were positive for IgG and IgM (40.9.3% and 24.2%, respectively) against at least one pathogen did not significantly differ (p=0.697 and p=0.188, respectively, Table 2) from those who sold domestic animal meat (40.4% and 10.6%, respectively) or only vegetables (48.6% and 18.9%, respectively). The overall prevalence of IgM positivity for *Leptospira* was 11.3% (95% CI: 6.9-17.8). Vendors selling wildlife and vegetables had higher proportion of STG IgG positivity (27.0% and 24.2%, respectively) than those selling domestic animal meat (6.4%, p=0.02). When looking at the effect of exposure to different items, STG IgG seropositivity was negatively associated with exposure to domestic animal meat (p<0.001) and positively with exposure to vegetables (p=0.001).

The presence of IgG against TG, STG and *Leptospira* spp. (based on IFA results for the former two) were tested across all three surveys (n=367). Overall, 32.7% (95% CI:25.4-40.9) of vendors were IgG seropositive for at least one pathogen at some point during the study. Twenty-three individuals (15.3%; 95% CI:10.2-22.3) sero-converted for at least one pathogen during the course of the study (i.e. the vendor seroconverted based on IgG levels transitioning from ‘negative’ to ‘positive’ in the proceeding survey) - five vegetable vendors, eight domestic animal meat vendors and ten wildlife meat vendors, but was not significant between vendor types (Fisher exact test, p=0.918) (Table 3 and Figure S1). Prevalence of anti-*Leptospira* IgG across all occasions was 12.0% (95% CI:7.5-18.6) and did not differ between vendor types (Chi-squared test, p=0.34). The prevalence of anti-STG IgG across occasions was 20% (95% CI:14.1-27.5) using ELISA and 12% (95% CI:7.5-18.6) using IFA and significantly differed between vendor types (Chi-Squared test, p=0.047), with vegetable and wildlife meat vendors (21.7%, 27.3% respectively) having higher prevalence than domestic animal meat vendors (8.5%) (not significant based on IFA results). Prevalence of anti-TG IgG was 24% (95% CI:17.6-31.8) using ELISA and 12.7% (95% CI:8.0-19.3) for IFA and did not significantly differ between vendor types (p=0.32) (Figure 2).

The best multi-season occupancy models for each pathogen are provided in supplementary Table S1. The best model for *Leptospira* spp. included the retail of domestic animal meat (as a binary variable). The fitted prevalence in individuals selling domestic animal meat or not were 11.6% (95% CI:6.1-21.0) and 4.5% (95% CI:1.5-13.3), respectively. This difference was not significant (p=0.5). In the second-best model (null model), estimated average prevalence was 8.4% (95% CI:4.8-14.3). The estimated incidence was 3.2% (95% CI:1.3-7.5) per 7.5-month period. The best model for STG included market and vendor type in the prevalence portion of the model. Prevalence in vegetable vendors and wildlife vendors (27.3%, 95% CI:15.3-43.8 and 28.4%, 95% CI:18.7-40.6, respectively) was significantly higher than the prevalence in domestic animal meat vendors (6.9 %, 95% CI:2.2-19.4; OR=0.24, 95% CI:0.06-1.0, p=0.05), and higher in the Xieng Khouang market (OR=9.6, 95% CI:1.2-78.4, p=0.03). Mean incidence was estimated at 0.57% (95% CI:0.022-13.1) per 7.5-month period. Finally, for TG, vendor age significantly affected prevalence (OR=1.04, 95% CI:1.01-1.08, p=0.006). Mean incidence was estimated at 0.64% (95% CI:0.088-4.51) per season (7.5-month period).

## Discussion

Previous work in markets in Laos have shown a high volume of wildlife meat trade [1, 15, 16]. In some communities, wildlife meat, including fish, may account for 66% of weekly protein intake [24]. With most wildlife vendors not perceiving risk from selling wildlife [15], we aimed to understand if any disease transmission was occurring in these markets and if it was associated with wildlife meat.

The relatively high prevalence of IgM positivity in Survey-1 (11.3%) was consistent with a high incidence of *Leptospira* sero-conversion. More vendors were IgM-positive for *Leptospira* than IgG-positive. The reasons for this are unclear, as Survey-1 was conducted before the wet season, prior to when we would expect leptospirosis outbreaks. Studies of immune responses to *Leptospira* spp. showed that not all patients produce IgG [25], and the IgM response was often higher and persisted longer than the IgG.

Analysis of samples from all three surveys showed fewer domestic animal meat vendors were positive for STG IgG than vegetable or wildlife meat vendors. As *O. tsutsugamushi* is transmitted by *Leptotrombidium* mites, and rodents are preferred hosts, we may expect wildlife meat vendors to be at greater risk of exposure. Unlike fleas, only 25-50% of *Leptotrombidium* mites will leave the carcass of a freshly killed mouse, and 60-75% would later reattach and continue feeding from a new host, although no time frame for detachment was given [26]. Therefore dead rodents may still harbour *Leptotrombidium* mites when being handled by vendors, which may result in increased risk of contact with live mites, that may attach to the vendor and therefore increase the risk of scrub typhus infection. In addition, vegetable sellers may more likely be exposed to rodents associated with agriculture [27], increasing risk of contact with mites and having an increased likelihood of coming into contact with mites in soil (either through farming or from soil attached to vegetables). As far as we know, there are no data on *Leptotrombidium* chiggers in vegetable-associated soil in markets. Given the high number of wildlife vendors also selling vegetables, our data did not allow separating the specific effects of wildlife meat and vegetables. The difference may be related to overall exposure patterns related to agricultural activities rather than one item in particular.

Vendors rarely hunted themselves, but instead purchased wildlife meat from hunters or via intermediaries [1]. Vectors for *R. typhi* (fleas such as *Xenopsylla cheopis)* will leave a dead host quickly due to body temperature cooling [28]. Therefore, wildlife meat vendors may be less exposed to fleas from dead animals, which could explain the absence of effect of vendor type on *R. typhi* sero-prevalence.

Direct contact with bodily fluids, through activities such as butchering meat, is the most significantly associated risk factor for seropositivity for zoonotic viruses such as henipaviruses [29, 30] and bacterial pathogens such as *Leptospira* spp. [31, 32]. Not only are *Leptospira* spp. one of the most common pathogens to be detected in dead wild animals sold at these markets [6], they can survive for prolonged periods in the environment [33]. Yet, no significant difference in *Leptospira* exposure between vendor types was seen. Whilst both *O. tsutsugamushi* and *R. typhi* are vector-borne pathogens, laboratory-acquired infections have occurred through puncture injuries [34], and injuries during the butchering process could conceivably result in infection without the need for a vector. However, this has not been documented. Reported injuries from wild animals was surprisingly high in this study (41.4%) compared to others; in Uganda, 14.6% of ‘traders’ reported an injury (including injuries from both wildlife and domesticated animals) [30].

There are a number of potential limitations with this study and interpretation of these data. Classification bias and the low number of seroconversion events may limit the statistical power of this analysis. Although repeat participation during the survey period was high,several vendors could not be contacted again. One reason is that one of the surveys coincided with increased agricultural activity. This may have introduced bias, as vendors whose livelihood relied on agricultural activities were more likely to be lost to follow-up. Although seasonality of the sale of wild animal meat has not been studied in Laos, a decrease in hunting and consumption during increased agricultural activity is seen in Africa, most likely due to availability of alternative incomes [35].

Despite relationships with market directors and vendors built up over several different studies at these sites [1, 16], not all vendors were forthcoming regarding wildlife selling and butchering. The trade in wildlife observed in markets is often illegal due to the protection status of the species traded, or that they have been harvested without adherence to regulations [36]. The low sample size for vendors selling single product types may have limited our ability to detect exposure via butchering and handling processes, highlighting the need for a greater resolution of human-animal contact activities, both for risks associated with their vending occupation (such as the butchering processes and exposure to urine), other activities associated with their daily lives, or other living conditions. In addition, no account was made for the quantity of item sold by the vendor, the animal species being sold, or how the vendor handled the wildlife (alive or dead). It is likely the greater the volume being sold, the greater the risk of infection.

## Conclusions

We have shown that vendors sero-converted for several endemic zoonotic bacterial pathogens. The seropositivity is suggestive of a link with what the vendor sells and warrants further investigation to untangle the web of transmission of zoonotic pathogens between wildlife and humans within and outside markets. The COVID-19 pandemic has highlighted the need to act to reduce the wildlife trade, especially for that catering to urban populations less impacted by food insecurity.

## Supplementary Material

Supplementary Material

## Figures and Tables

**Table 1:** Sample and demographic data of market vendors enrolled in study. *Market sites are: BK = Bolikhamxay; SV = Salavan; XK = Xieng Khouang.

		Market sites*			All markets	p-value
		BK	SV	XK		
No of vendors	Survey 1	31	61	58	150	
	Survey 2	26	41	50	117	
	Survey 3	26	34	40	100	
No of samples from each vendor	1 sample	4	19	8	31	
	2 samples	2	9	10	21	
	3 samples	25	33	40	98	
Total samples collected		83	136	148	367	
Gender (female)		31/31 (100%)	61/61 (100%)	58/58 (100%)	150/150 (100%)	
Median age (years)		40 (n = 31) IQR: 32-47	36 (n = 61) IQR: 29-45	40 (n = 58) IQR: 33-48	38 (n = 150) IQR: 30-46	0.281
Level of education	Illiterate	6/31 (19.4%)	18/61 (29.5%)	3/58 (5.2%)	27/150 (18.0%)	<0.001
	Primary	14/31 (45.2%)	25/61 (41.0%)	12/58 (20.7%)	51/150 (34.0%)	
	Secondary	10/31 (32.3%)	17/61 (27.9%)	41/58 (70.7%)	68/150 (45.3%)	
	Professional school or university	1/31 (3.2%)	1/61 (1.6%)	2/58 (3.4%)	4/150 (2.7%)	
Ethnic group	Lao Loum	19/31 (61.3%)	32/61 (52.5%)	47/58 (81.0%)	98/150 (65.3%)	<0.001
	Hmong	6/31 (19.4%)	0/61 (0.0%)	7/58 (12.1%)	13/150 (8.7%)	
	Mon-Khmer	0/31 (0.0%)	0/61 (0.0%)	1/58 (1.7%)	1/150 (0.6%)	
	Ta-Oy	0/31 (0.0%)	6/61 (9.8%)	0/58 (0.0%)	6/150 (4.0%)	
	Ngae	0/31 (0.0%)	8/61 (13.1%)	0/58 (0.0%)	8/150 (5.3%)	
	Other	6/31 (19.4%)	15/61 (24.6%)	3/58 (5.2%)	24/150 (16.0%)	
Median length of time as a vendor (years)		10 (n = 31) IQR: 4-11	5 (n = 61) IQR: 3-10	10 (n = 58) IQR: 6-10	7 (n = 150) IQR: 4-10	0.118

**Table 2:** IgM and IgG positivity by ELISA of market vendors from the first survey (Survey-1). STG = scrub typhus group; TG = typhus group. Vendor type: V_o_ = vegetables only; DM_v_ = domestic animal meat +/- vegetables; WM_v/dm_ = wildlife meat +/- vegetables, +/- domestic animalmeat

Vendor type		All		V_o_		DM_v_		WM_v/dm_		*p-value*
		*n*	%	*n*	%	*n*	%	*n*	%	
Total		150		37		47		66		
All	IgM	28	18.7	7	18.9	5	10.6	16	24.2	0.188
	IgG	64	42.7	18	48.6	19	40.4	27	40.9	0.697
STG	IgM	10	6.7	2	5.4	2	4.3	6	9.1	0.561
	IgG	29	19.3	10	27.0	3	6.4	16	24.2	0.024
TG	IgM	4	2.7	1	2.7	0	0.0	3	4.5	0.335
	IgG	37	24.7	11	29.7	14	29.8	12	18.2	0.264
*Leptospira* spp.	IgM	17	11.3	5	13.5	3	6.4	9	13.6	0.434
	IgG	9	6.0	1	2.7	3	6.4	5	7.6	0.602

**Table 3:** Number of vendors, with two or more samples, who seroconverted based on IgG levels transitioning from ‘negative’ to ‘positive’ in the proceeding survey. STG = scrub typhus group; TG = typhus group. Vendor type: VO = vegetables only; DMV = domestic animal meat +/- vegetables; WM_v/dm_ = wildlife meat +/- vegetables, +/- domestic animal meat

	V_o_	DM_v_	WM_v/dm_
STG	1/25	2/42	3/52
TG	4/25	4/42	4/52
*Leptospira* spp.	1/25	3/42	3/52

## References

[1] Pruvot M, Khammavong K, Milavong P, Philavong C, Reinharz D, Mayxay M, Rattanavong S, Horwood P, Dussart P, Douangngeun B, Theppangna W (2019). Toward a quantification of risks at the nexus of conservation and health: The case of bushmeat markets in Lao PDR. Sci Total Environ.

[2] Krishnasamy K, Zavagli M (2020). Southeast Asia at the heart of wildlife trade.

[3] Organisation for Economic Co-operation and Development (OECD) (2019). The Illegal Wildlife Trade in Southeast Asia.

[4] Shairp R, Verissimo D, Fraser I, Challender D, MacMillan D (2016). Understanding Urban Demand for Wild Meat in Vietnam: Implications for Conservation Actions. PLoS One.

[5] Bennett EL (2002). Is There a Link between Wild Meat and Food Security?. Conservation Biology.

[6] Nawtaisong P, Robinson MT, Khammavong K, Milavong P, Rachlin A, Dittrich S, Dubot-Pérès A, Vongsouvath M, Horwood PF, Dussart P, Theppangna W (2022). Zoonotic pathogens in wildlife traded in markets for human consumption, Laos. Emerg Infect Dis.

[7] Taylor LH, Latham SM, Woolhouse ME (2001). Risk factors for human disease emergence. Philos Trans R Soc Lond B Biol Sci.

[8] Jones KE, Patel NG, Levy MA, Storeygard A, Balk D, Gittleman JL, Daszak P (2008). Global trends in emerging infectious diseases. Nature.

[9] Kurpiers LA, Schulte-Herbrüggen B, Ejotre I, Reeder DM, Angelici F (2016). Problematic Wildlife.

[10] Grace D, Gilbert J, Randolph T, Kang’ethe E (2012). The multiple burdens of zoonotic disease and an Ecohealth approach to their assessment. Trop Anim Health Prod.

[11] Shi Z, Hu Z (2008). A review of studies on animal reservoirs of the SARS coronavirus. Virus Res.

[12] Reina J (2020). The SARS-CoV-2, a new pandemic zoonosis that threatens the world. Vacunas (English Edition).

[13] Mari Saez A, Weiss S, Nowak K, Lapeyre V, Zimmermann F, Dux A, Kuhl HS, Kaba M, Regnaut S, Merkel K, Sachse A (2015). Investigating the zoonotic origin of the West African Ebola epidemic. EMBO Mol Med.

[14] Phongmany S, Rolain J-M, Phetsouvanh R, Blacksell SD, Soukkhaseum V, Rasachack B, Phiasakha K, Soukkhaseum S, Frichithavong K, Chu V, Keolouangkhot V (2006). Rickettsial infections and fever, Vientiane, Laos. Emerg Infect Dis.

[15] Philavong C, Pruvot M, Reinharz D, Mayxay M, Khammavong K, Milavong P, Rattanavong S, Horwood PF, Dussart P, Douangngeun B, Theppangna W (2020). Perception of health risks in Lao market vendors. Zoonoses Public Health.

[16] Greatorex ZF, Olson SH, Singhalath S, Silithammavong S, Khammavong K, Fine AE, Weisman W, Douangngeun B, Theppangna W, Keatts L, Gilbert M (2016). Wildlife trade and human health in Lao PDR: an assessment of the zoonotic disease risk in markets. PLoS One.

[17] Phanichkrivalkosil M, Tanganuchitcharnchai A, Jintaworn S, Kantipong P, Laongnualpanich A, Chierakul W, Paris DH, Richards AL, Wangrangsimakul T, Day NPJ, Blacksell SD (2019). Determination of Optimal Diagnostic Cut-Offs for the Naval Medical Research Center Scrub Typhus IgM ELISA in Chiang Rai, Thailand. Am J Trop Med Hyg.

[18] Blacksell SD, Lim C, Tanganuchitcharnchai A, Jintaworn S, Kantipong P, Richards AL, Paris DH, Limmathurotsakul D, Day NPJ (2016). Optimal Cutoff and Accuracy of an IgM Enzyme-Linked Immunosorbent Assay for Diagnosis of Acute Scrub Typhus in Northern Thailand: an Alternative Reference Method to the IgM Immunofluorescence Assay. Journal of Clinical Micrbiology.

[19] Blacksell SD, Jenjaroen K, Phetsouvanh R, Tanganuchitcharnchai A, Phouminh P, Phongmany S, Day NP, Newton PN (2010). Accuracy of rapid IgM-based immunochromatographic and immunoblot assays for diagnosis of acute scrub typhus and murine typhus infections in Laos. Am J Trop Med Hyg.

[20] Phakhounthong K, Mukaka M, Dittrich S, Tanganuchitcharnchai A, Day NPJ, White LJ, Newton PN, Blacksell SD (2020). The temporal dynamics of humoral immunity to *Rickettsia typhi* infection in murine typhus patients. Clin Microbiol Infect.

[21] MacKenzie D, Nichols JD, Royle JA, Pollock KH, Bailey LL, Hines JE (2017). Occupancy Estimation and Modeling: Inferring Patterns and Dynamics of Species Occurrence.

[22] Bailey LL, MacKenzie DI, Nichols JD (2014). Advances and applications of occupancy models. Methods in Ecology and Evolution.

[23] MacKenzie D, Hines J (2021). RPresence: R interface for program PRESENCE, R package, version 21310.

[24] Johnson A, Singh S, Dongdala M, Vongsa O (2003). Wildlife hunting and use in the Nam Ha National Protected Area, Lao PDR: implications for rural livelihoods and biodiversity conservation.

[25] Adler B, Faine S (1978). The antibodies involved in the human immune response to leptospiral infection. J Med Microbiol.

[26] Traub R, Wisseman CL, Jones MR, O’Keefe JJ (1975). The acquisition of *Rickettsia tsutsugamushi* by chiggers (trombiculid mites) during the feeding process. Ann N Y Acad Sci.

[27] Singleton GR, Belmain S, Brown PR, Aplin K, Htwe NM (2010). Impacts of rodent outbreaks on food security in Asia. Wildlife Research.

[28] Feldhamer GA, Drickamer LC, Vessey SH, Merritt JF, Krajewski C (2007). Mammalogy: adaptation, diversity, ecology.

[29] Pernet O, Schneider BS, Beaty SM, LeBreton M, Yun TE, Park A, Zachariah TT, Bowden TA, Hitchens P, Ramirez CM, Daszak P (2014). Evidence for henipavirus spillover into human populations in Africa. Nat Commun.

[30] Paige SB, Frost SD, Gibson MA, Jones JH, Shankar A, Switzer WM, Ting N, Goldberg TL (2014). Beyond bushmeat: animal contact, injury, and zoonotic disease risk in Western Uganda. Ecohealth.

[31] Feuer B, Domash-Martinez T (2011). Report of case: leptospirosis after exposure to alligator carcass. Osteopathic Family Physician.

[32] Dorjee S, Heuer C, Jackson R, West DM, Collins-Emerson JM, Midwinter AC, Ridler AL (2011). Assessment of occupational exposure to leptospirosis in a sheep-only abattoir. Epidemiol Infect.

[33] Casanovas-Massana A, Pedra GG, Wunder EA, Diggle PJ, Begon M, Ko AI (2018). Quantification of Leptospira interrogans Survival in Soil and Water Microcosms. App Environ Microbiol.

[34] Blacksell SD, Robinson MT, Newton PN, Day NPJ (2019). Laboratory-acquired scrub typhus and murine typhus infections: the argument for a risk-based approach to biosafety requirements for *Orientia tsutsugamushi* and *Rickettsia typhi* laboratory activities. Clin Infect Dis.

[35] Brashares JS, Golden CD, Weinbaum KZ, Barrett CB, Okello GV (2011). Economic and geographic drivers of wildlife consumption in rural Africa. Proc Natl Acad Sci U S A.

[36] Knapp EJ, Rentsch D, Schmitt J, Lewis C, Polasky S (2010). A tale of three villages: choosing an effective method for assessing poaching levels in western Serengeti. Tanzania, Oryx.

