# Supplementary material for: Longitudinal comparison of bacterial pathogen seropositivity among wet market vendors in the Lao People's Democratic Republic
Source: One Health. 2023 Aug 22;17:100618. doi: 10.1016/j.onehlt.2023.100618 (PMC7615163; doi:10.1016/j.onehlt.2023.100618)
Supplement: Heat map of seropositivity of market vendors over survey period and table of the best multi-season occupancy models for each pathogen studied [file mmc1.docx]

**Supplementary figure S1.** Heat map of seropositivity of market vendors over survey period for scrub typhus group (STG) and typhus group (TG) IgG by IFA, and *Leptospira* spp. IgG ELISA, separated by market. Each column represents a single market vendor, grouped by vendor type: V_O_ = vegetables only (no meat sold), DM_V_ = domestic animal meat (+/- vegetables), WM_V/DM_ = wildlife meat (+/- vegetables, +/- domestic animal meat).


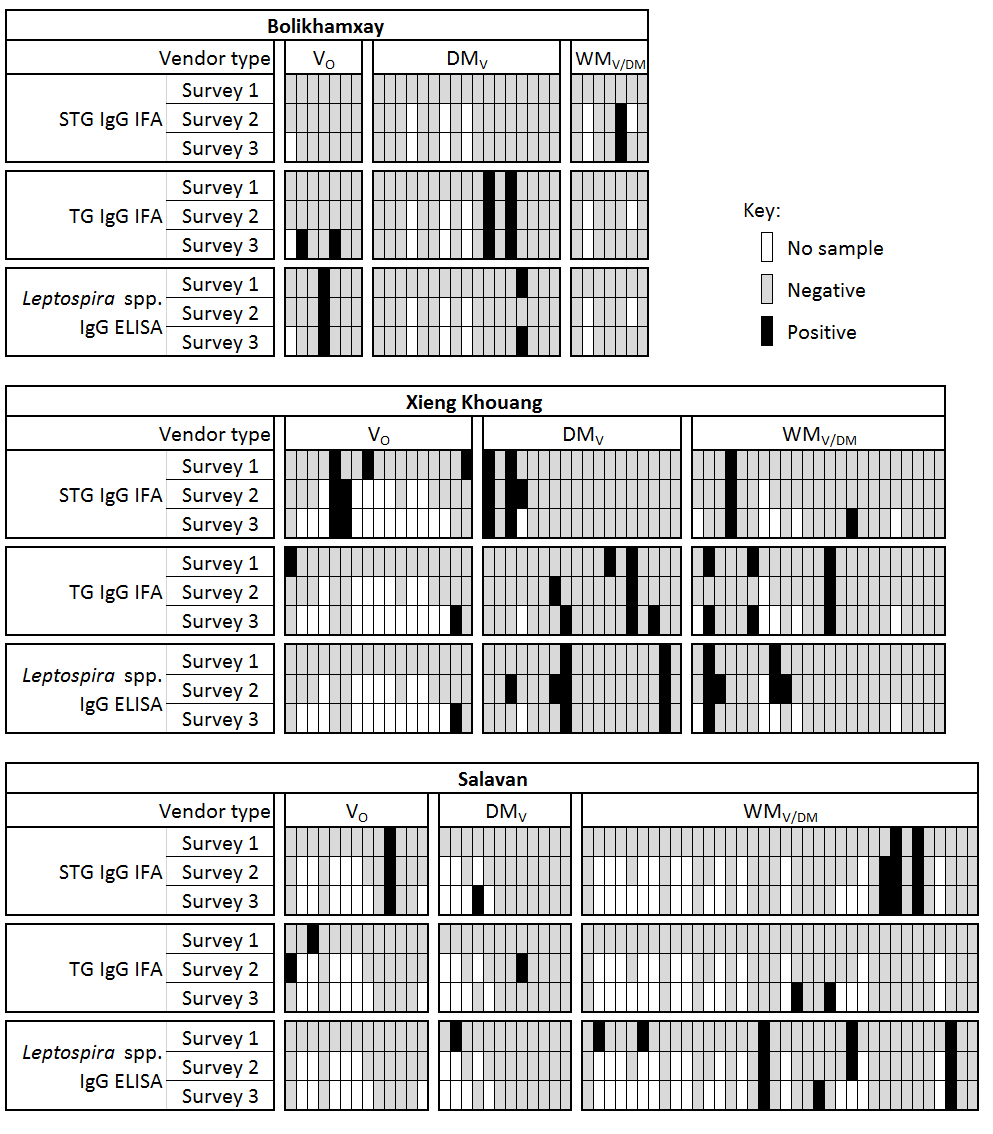


**Supplementary table S2.**

The best multi-season occupancy models for each pathogen studied. AIC = Akaike information criterion. k = number of parameters in the model.

| Outcome | Model | AIC | k |
| --- | --- | --- | --- |
| Leptospira ELISA IgG | psi(fm)gamma(.)epsilon(.)p(.) | 186 | 5 |
| Scrub Typhus Group ELISA IgG | psi(market + type)gamma(.)epsilon(.)p(.) | 263.3 | 8 |
| Typhus Group ELISA IgG | psi(age)gamma(.)epsilon(.)p(.) | 243.8 | 5 |
