# Supplementary material for: Longitudinal comparison of bacterial pathogen seropositivity among wet market vendors in the Lao People's Democratic Republic
Source: One Health. 2023 Aug 22;17:100618. doi: 10.1016/j.onehlt.2023.100618 (PMC7615163; doi:10.1016/j.onehlt.2023.100618)
Supplement: Questionnaire (english language version) given to market vendors at each survey point. [file mmc2.pdf]

Questionnaire for vegetable, domestic meat, wildlife meat vendors.

1. Date of Visit: __ __/__ __/2017 (JJ/MM/2017)
2. Nameof Investigator: [redacted]
3. Province Code : __ __/ (1=XK, 2=LAK 20, 3=SRV)
4. Participant code: __ __ __ __ (Study site/No. of patients in the market)

Code P

I. Socio-economic and demographic characteristics of household:

1	Age: __ __ years	Year of birth __ __ __ __
2	Gender	❑0 Female ❑ 1Male
3	Ethnic group	❑ 1 Lao ❑ 2 Hmong ❑3 Mon-Khmer ❑ 4 Ta-Oy<br>❑ 5 Ngae ❑ 6 other……
4	Level of educationcompleted	❑ 1 Illiterate ❑ 2Primary ❑3 Secondary ❑ 4Professional school or university
5	Which kind of vendor	❑1Vegetable only<br>❑2 Domestic meat only<br>❑3 Wildlife meat only<br>❑4 Vegetable + domestic meat<br>❑5 Vegetable + wildlife meat<br>❑6Domestic + wildlife meat<br>❑7 etc.
6	Length of being vendors	…………..year<br>Vegetable only (jump to II)<br>Domestic meat only (jump to III)<br>Wildlife meat only (jump to IV)
**II.**	**For vegetable sellers**	
7	Have you been selling wildlife or deomestic meat	❑0No (jump to 16) ❑ 1yes if yes, which ones<br>(If domestic go to III, if wildlife go to wildlife IV, if both go to III and IV)
8	Are you famer ?	❑0No❑ 1yes (go to take blood)
**III.**	**For domestic meat sellers**	
9	Do you have domestic animals at home	❑0No❑ 1yes if yes which ones…..<br>(Jump to 15)
10	Do you butcher animals yourself ?	
**IV.**	**For wildlife sellers**	
11	Do you have wildlife animals at home?	❑0No❑ 1yes If yes, which one……

12	Are you a hunter?	❑$_0$No❑ $_1$yes If yes, since when……
14	Do you hunt for food or for sale ?	❑$_0$No❑ $_1$yes
15	Do you sell these animals at the market?	❑$_0$No❑ $_1$yes
16	Do you butcher animals whose meat is sold at the market?	❑$_0$No❑ $_1$yes
17	Do you butcher animals for personal food consumption ?	❑$_0$No❑ $_1$yes
18	Have you eve had an injury form wildlife biting/scratching ?	❑$_0$No❑ $_1$yes

Examination & Blood Analysis

**Study Site (market)**			
**Date of Test**			
**Examinator ID:**			
Participant sitting for 15 minutes before 1st measurement; 3 minutes between measurements	**Completed**<br>**(X) (time hh:mm)**	**Systolic blood pressure (mm Hg)**	**Diastolic blood pressure (mm Hg)**
**BP Measurement :**			
**Blood test**	**Completed**<br>**(X)**	**Result blood test**	
**Glucose test**			
**Blood tube**			
