# Supplementary material for: Longitudinal comparison of bacterial pathogen seropositivity among wet market vendors in the Lao People’s Democratic Republic
Source: One Health. Author manuscript; Available in PMC 2023 Dec 1. (PMC7615163; doi:10.1016/j.onehlt.2023.100618)
Supplement: Supplementary file 1 [file EMS185184-supplement-Supplementary_Material.pdf]

Supplementary figure S1. Heat map of seropositivity of market vendors over survey period for scrub typhus group (STG) and typhus group (TG) IgG by IFA, and *Leptospira* spp. IgG ELISA, separated by market. Each column represents a single market vendor, grouped by vendor type: V_O = vegetables only (no meat sold), DM_V = domestic animal meat (+/- vegetables), $WM_{V/DM}$ = wildlife meat (+/- vegetables, +/- domestic animal meat).

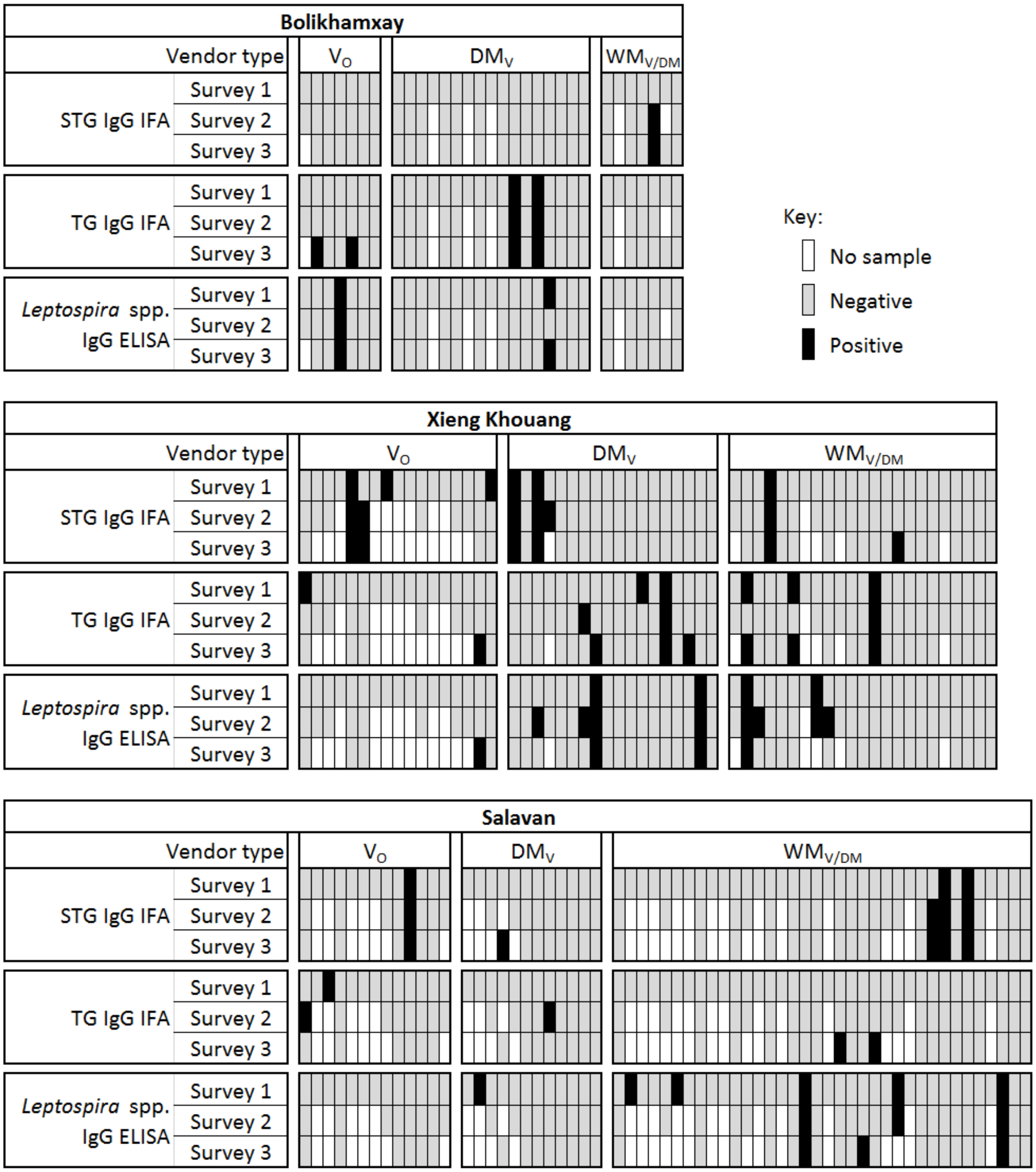

Supplementary table S2.

The best multi-season occupancy models for each pathogen studied. AIC = Akaike information criterion. k = number of parameters in the model.

Outcome	Model	AIC	k
Leptospira ELISA IgG	psi(fm)gamma(.)epsilon(.)p(.)	186	5
Scrub Typhus Group ELISA IgG	psi(market + type)gamma(.)epsilon(.)p(.)	263.3	8
Typhus Group ELISA IgG	psi(age)gamma(.)epsilon(.)p(.)	243.8	5

Questionnaire for vegetable, domestic meat, wildlife meat vendors.

1. Date of Visit: __ __/__ __/2017 (JJ/MM/2017)
2. Nameof Investigator: [redacted]
3. Province Code : __ __/ (1=XK, 2=LAK 20, 3=SRV)
4. Participant code: __ __ __ __ (Study site/No. of patients in the market)

Code P

I. Socio-economic and demographic characteristics of household:

1	Age: __ __ years	Year of birth __ __ __ __
2	Gender	❑$_0$ Female ❑ $_1$Male
3	Ethnic group	❑ $_1$ Lao ❑ $_2$ Hmong ❑$_3$ Mon-Khmer ❑ $_4$ Ta-Oy<br>❑ $_5$ Ngae ❑ $_6$ other……
4	Level of educationcompleted	❑ $_1$ Illiterate ❑ $_2$Primary ❑$_3$ Secondary ❑ $_4$Professional school or university
5	Which kind of vendor	❑$_1$Vegetable only<br>❑$_2$ Domestic meat only<br>❑$_3$ Wildlife meat only<br>❑$_4$ Vegetable + domestic meat<br>❑$_5$ Vegetable + wildlife meat<br>❑$_6$Domestic + wildlife meat<br>❑$_7$ etc.
6	Length of being vendors	…………..year<br>Vegetable only (jump to II)<br>Domestic meat only (jump to III)<br>Wildlife meat only (jump to IV)
**II.**	**For vegetable sellers**	
7	Have you been selling wildlife or deomestic meat	❑$_0$No (jump to 16) ❑ $_1$yes if yes, which ones<br>(If domestic go to III, if wildlife go to wildlife IV, if both go to III and IV)
8	Are you famer ?	❑$_0$No❑ $_1$yes (go to take blood)
**III.**	**For domestic meat sellers**	
9	Do you have domestic animals at home	❑$_0$No❑ $_1$yes if yes which ones…..<br>(Jump to 15)
10	Do you butcher animals yourself ?	
**IV.**	**For wildlife sellers**	
11	Do you have wildlife animals at home?	❑$_0$No❑ $_1$yes If yes, which one……

[redacted], [redacted] [redacted]

12	Are you a hunter?	❑ $_0$No ❑ $_1$yes If yes, since when……
14	Do you hunt for food or for sale ?	❑ $_0$No ❑ $_1$yes
15	Do you sell these animals at the market?	❑ $_0$No ❑ $_1$yes
16	Do you butcher animals whose meat is sold at the market?	❑ $_0$No ❑ $_1$yes
17	Do you butcher animals for personal food consumption ?	❑ $_0$No ❑ $_1$yes
18	Have you eve had an injury form wildlife biting/scratching ?	❑ $_0$No ❑ $_1$yes

Examination & Blood Analysis

**Study Site (market)**			
**Date of Test**			
**Examinator ID:**			
Participant sitting for 15 minutes before 1st measurement; 3 minutes between measurements	**Completed (X) (time hh:mm)**	**Systolic blood pressure (mm Hg)**	**Diastolic blood pressure (mm Hg)**
**BP Measurement :**			
**Blood test**	**Completed (X)**	**Result blood test**	
**Glucose test**			
**Blood tube**			

[redacted] [redacted]
